# Supplementary material for: MetaRibo-Seq measures translation in microbiomes
Source: Nat Commun. 2020 Jun 29;11:3268. doi: 10.1038/s41467-020-17081-z (PMC7324362; doi:10.1038/s41467-020-17081-z)
Supplement: Supplementary file 10 — Supplementary Data 7 [file 41467_2020_17081_MOESM10_ESM.zip › File2/Confidence_High_Taxonomy/183236_out.krona.html]

Javascript must be enabled to view this page.

members
magnitude
magnitudeUnassigned
count
unassigned
taxon
rank

183236\_out

16

16
superkingdom
2

16
phylum
1239

class
16
186801

order
16
4
186802

SRS144297\_contig\_number\_contig-100\_6071.83222SRS147139\_contig\_number\_contig-100\_2446.76470SRS150029\_contig\_number\_contig-100\_4712.96359SRS893327\_contig\_number\_contig-100\_3084.70282

2
family
186803

genus
1
572511

1

SRS043701\_contig\_number\_7707
species
418240

1
genus
841


SRS024075\_contig\_number\_11578
species
1
360807

541000
10
family

1263

SRS017191\_contig\_number\_15611SRS077552\_contig\_number\_25584SRS097889\_contig\_number\_contig-100\_4110.132676SRS1041090\_contig\_number\_1856SRS144183\_contig\_number\_21131
genus
5
10

2293224
species

SRS020233\_contig\_number\_5596
1

species

SRS018817\_contig\_number\_2106SRS143342\_contig\_number\_contig-100\_8823.86024
2
2293192


SRS1041091\_contig\_number\_20547
species
1
2293198

2293225

SRS893270\_contig\_number\_11134
species
1
